# Development of a molecular marker for the Run8 gene for the selection of barley genotypes resistant to smut

**DOI:** 10.18699/vjgb-26-41

**Published:** 2026-05

**Authors:** E.A. Orlova, N.P. Bechtold, Yu.N. Grigoriev, O.Yu. Shoeva, A.Yu. Glagoleva, T.V. Kukoeva

**Affiliations:** Siberian Research Institute of Plant Production and Breeding – Branch of the Institute of Cytology and Genetics of the Siberian Branch of the Russian Academy of Sciences, Krasnoobsk, Novosibirsk region, Russia; Siberian Research Institute of Plant Production and Breeding – Branch of the Institute of Cytology and Genetics of the Siberian Branch of the Russian Academy of Sciences, Krasnoobsk, Novosibirsk region, Russia; Siberian Research Institute of Plant Production and Breeding – Branch of the Institute of Cytology and Genetics of the Siberian Branch of the Russian Academy of Sciences, Krasnoobsk, Novosibirsk region, Russia; Institute of Cytology and Genetics of the Siberian Branch of the Russian Academy of Sciences, Novosibirsk, Russia; Institute of Cytology and Genetics of the Siberian Branch of the Russian Academy of Sciences, Novosibirsk, Russia; Institute of Cytology and Genetics of the Siberian Branch of the Russian Academy of Sciences, Novosibirsk, Russia

**Keywords:** barley, loose smut, resistance genes, markers, lines, ячмень, пыльная головня, гены устойчивости, маркеры, линии

## Abstract

Loose smut of barley, caused by the basidiomycete Ustilago nuda (Jens.) Roster, occurs in all regions of the world where this crop is grown. This seed-borne disease causes significant losses in grain production. Selection for resistance to loose smut based on the use of donors with resistance genes is an ecologically and economically safe way to constrain the negative impact of the pathogen on barley. The introduction of molecular genetic approaches into the breeding process makes it possible to control the transfer of resistance genes to hybrid material. The Run8 gene controls resistance to many isolates of loose smut, including in the West Siberian region of Russia. The objective of the current study is to develop a molecular marker for Run8 for the selection of barley genotypes resistant to loose smut from hybrid populations. By comparing the nucleotide sequences of the Run8 gene available from the barley pangenome database, an insertion/deletion of six nucleotide pairs in the coding region of the gene was identified. Based on the identified polymorphism, a molecular marker Hor7050 was developed, which allows differentiating the alleles of Run8. The developed marker was tested on hybrid lines (F5–F6) obtained from crossing cultivar Elf, which is a donor of resistance to loose smut and carries, according to the originators, the Run8 gene, with cultivar Tanai, which has practical resistance to the pathogen. Using the developed marker, 18 hybrids carrying Run8 of Elf were selected from 84 hybrids; however, the phytopathological assessment showed that eight of the selected lines were susceptible to the disease. To clarify the genotype of 18 selected lines, an additional analysis was carried out using the microsatellite marker EBmac0541 linked to Run6. A relationship was established between the presence of the allele of this marker from Elf and resistance to the disease. It is possible that Elf, in addition to Run8, carries Run6, which is effective against race 1 of the causative agent of loose smut. Additional studies are required to clarify the presence of Run6 in the Elf variety. In addition to resistance, the selected lines were characterized by productivity traits. According to the two-year analysis, three productive resistant lines were identified, with Run8 – 32, 65 and 79, significantly exceeding the control Elf in yield. The selected lines were transferred to breeding nurseries for their further evaluation by economically important traits.

## Abstract (ru)

Пыльная головня ячменя, вызываемая базидиомицетом Ustilago nuda (Jens.) Roster, встречается во всех регионах, где возделывается эта культура. Селекция на резистентность к пыльной головне, основанная на использовании доноров, обладающих генами устойчивости, представляет экологически и экономически безопасный способ, сдерживающий отрицательное действие патогена на ячмень. Внедрение в селекционный процесс молекулярно-генетических подходов позволяет контролировать передачу генов резистентности в гибридный материал. Ген Run8 контролирует устойчивость ко многим изолятам пыльной головни и служит источником высокой устойчивости к этому возбудителю. Цель представленной работы – разработка молекулярного маркера к гену Run8 для отбора из гибридных популяций генотипов ячменя, устойчивых к пыльной головне. С помощью сравнения доступных из базы данных пангенома ячменя нуклеотидных последовательностей гена Run8 определена инсерция/делеция шести пар нуклеотидов в кодирующей области гена. На основе выявленного полиморфизма разработан молекулярный маркер Hor7050, позволяющий дифференцировать аллели гена Run8. Маркер был протестирован на гибридных линиях F5–F6, полученных от скрещивания сорта Эльф – донора резистентности к пыльной головне – и несущего, по данным оригинаторов, ген Run8, с сортом Танай, обладающим практической устойчивостью к возбудителю. С помощью разработанного маркера из 84 гибридов были отобраны 18, несущих ген Run8 сорта Эльф. Однако проведенная фитопатологическая оценка показала, что из выделенных линий восемь восприимчивы к заболеванию. Для уточнения генотипа 18 отобранных линий был проведен дополнительный анализ с помощью микросателлитного маркера EBmac0541, сцепленного с геном Run6. Была установлена взаимосвязь между наличием аллеля данного маркера от сорта Эльф и устойчивостью к заболеванию. Возможно, сорт Эльф, кроме гена Run8, несет ген Run6, эффективный против расы 1 возбудителя пыльной головни. Для уточнения наличия гена Run6 у сорта Эльф требуются дополнительные исследования. Помимо устойчивости, отобранные линии были охарактеризованы по признакам продуктивности. По результатам двухлетнего анализа выделены три продуктивные резистентные линии с геном Run8 – 32, 65 и 79, достоверно превышающие по урожайности контрольный сорт Эльф.

## Introduction

Barley is one of the main forage cereals. Its high plasticity and short growing season allow it to be cultivated in a wide range of soil and climatic zones. Modern barley breeding is aimed at developing high-yielding varieties resistant to biotic and abiotic stress factors. Diseases caused by phytopathogenic fungi, with loose smut being the most widespread and harmful, hinder the achievement of high and stable yields

Loose smut of barley, caused by the basidiomycete Ustilago nuda (Jens.) Roster occurs in all regions of the world where this crop is grown (Nielsen, Thomas, 1996). This seed-borne disease causes significant losses in grain production. When infected by the pathogen, a smut sorus containing teliospores forms instead of a spike. Thus, the damage from loose smut is proportional to the percentage of infected spikes in the field (Druzhin, Krupnov, 2008). However, economic losses are significantly higher. According to many authors, the pathogen has a suppressive effect at all stages of plant development. As a result of seedling death, field germination rate decreases; loose smut negatively affects the number of fertile tillers per plant, plant height, main spike length, number of grains per spike, and 1,000 grain weight (Orlova et al., 2015; Usoltsev, 2018). Most spring barley varieties included in the State Register of Varieties and Hybrids approved for use in the Russian Federation (https://gossortrf.ru/registry), are susceptible to loose smut to varying degrees. Control of the disease involves mandatory seed treatment with systemic fungicides. Therefore, cultivation of resistant varieties is preferable from an economic standpoint. On top of that, the value of resistant varieties is that they can be used in low-input organic farming systems to produce environmentally safe products without additional protective measures.

Throughout the years, many researchers have confirmed that genetic resistance of spring barley to loose smut can be controlled by individual dominant or several recessive genes (Krivchenko, 1984; Menzies et al., 2010). The loose smut resistance gene Run was first identified by J.E. Livingston (1942) in the variety Trebi. Later, he also identified a weak resistance gene Run2 in Missuri variety. W.P. Skoropad and L.P. Johnson (1952) identified two independent dominant genes Run3 and Run6 in Jet variety. The latter was used as the base for the Keystone variety carrying the Run6 gene. C.W. Schaller (1949) identified two dominant genes Run4 and Run5 in barley variety Dorsett and X173-10-5-6 hybrid. In Anоidium cultivar, the resistance is controlled by the recessive gene run7. The Run8 gene was isolated by D.R. Metcalfe (1966) from the winter barley line C.I. 4966 introduced to the United States from Russia. To date, 15 resistance genes to the loose smut pathogen have been identified in barley (Zang et al., 2015; Legkun et al., 2016), but according to the literature, only three of them (Run3, Run6, and Run8) are effective. The remaining genes are of low effectiveness and have not found practical use in breeding (Krivchenko, 1984).

Assessment of barley varieties for resistance to loose smut under natural infection conditions is not always reliable, which can lead to incorrect classification of varieties by resistance level. On the other hand, artificial inoculation is a labor-intensive and time-consuming process that spans two growing seasons as follows: in the first year, spikes are inoculated with a pathogen suspension and mature seeds are collected; in the following year, plants grown from the inoculated seeds are evaluated for resistance based on phenotypic expression. Moreover, the quality of infection largely depends on meteorological conditions during inoculation. According to L.V. Meshkova (2024), there is a high correlation between moisture, precipitation, and infection (r > 0.7), and a moderate negative correlation with air temperature (r = –0.44). Therefore, an effective method to develop resistant varieties is to combine the classical approaches based on hybridization of resistance gene donors with recipient varieties and modern molecular marker-aided breeding methods. This approach helps reduce the time and volume of unpromising hybrid material in the breeding process.

To determine the resistance of samples, it is necessary to consider the prevalent pathogen races in the target cultivation zone of future varieties. This is the only way to ensure staying one step ahead of the pathogen in the development of resistant genotypes. Understanding the genetics of resistance in the context of pathogen virulence makes it possible to identify the effective genes, for which geneticists can select DNA markers necessary for developing hybrids with high disease resistance based on genotype. The Run8 gene confers resistance to most races of the loose smut pathogen and is widely used in barley breeding both in Russia and abroad. This gene was mapped to the long arm of chromosome 1H (Zang et al., 2015). It codes for a protein containing two tandem protein kinase domains, which, as recently discovered, represent an important class of proteins involved in plant resistance. Polymorphism in the Run8 protein sequences has been identified, which allows the development of intragenic diagnostic molecular markers for precise identification of Run8 gene alleles. Before the nucleotide sequence of the Run8 gene was discovered, molecular markers linked to this gene were used in scientific research and breeding practice (Li et al., 2001; Eckstein et al., 2002). However, the diagnostic efficiency of the latter is lower than that of intragenic markers due to recombination between the linked markers and the target gene. Therefore, the development of intragenic molecular markers based on the known nucleotide sequences of target genes becomes a relevant research problem. The goal of the present paper was to develop an intragenic molecular marker for the Run8 gene and use it in combination with a marker linked to the Run6 gene for genotyping barley samples, so that the lines with effective resistance genes demonstrating high loose smut resistance could be singled out from hybrid populations.

## Materials and methods

**Plant material.** The study material consisted of 84 breeding lines selected in the F5–F6 generations from the Elf × Tanai cross. Elf variety is highly resistant to loose smut with the pedigree as follows: Roland × 1325 line, the latter being a dihaploid (Pervenets × Zazersky 85) F1 × H. Bulbosum, which carries the Run8 resistance gene presumably inherited from the Pervenets variety, according to the variety originators (State Register of Protected Selection Achievements). Tanai variety exhibits practical resistance to the pathogen and was developed from two breeding lines, G 20275 × G 20191, with Jet (Run3 Run6) and Paragon (Run3) varieties present in the pedigree.

The research was conducted from 2021 to 2023. In 2021, the 84 test lines were grown under field conditions in the SP1 breeding nursery. The lines selected for resistance were then reinoculated and sown in the phytopathological field of SibRIPP&B – Branch of IC&G SB RAS.

**Differentiation of the loose smut pathogen U. nuda.** set of differential varieties and a genetic set of test varieties with known resistance genes proposed by V.I. Krivchenko (1984) were used. Race identification was performed by comparing the reaction of differential varieties with a key for determining physiological races. The virulence formula of the race was determined based on the susceptibility of the genetic test set. Resistance reactions were classified according to the international scale: R standing for resistance (infection up to 10 %); S for susceptibility (infection above 10 %).

**Assessment of line resistance to loose smut.** Lines with confirmed resistance genes and the parental varieties Elf and Tanai were inoculated in June 2021 with the Novosibirsk population of loose smut. The susceptible indicator variety Grace was used to control inoculation quality. In 2022, the lines that showed resistance were reinoculated. Inoculation of barley spikes (at least eight per line) was carried out using the vacuum method during the early flowering stage, according to the VIR methodology (Sanin et al., 2008). The spore suspension was prepared immediately before inoculation at a concentration of 1 g of spores per 1 l of water. Smut spores were collected from infected spikes of various barley genotypes. To determine the race composition of the pathogen, differential and genetic test sets of barley varieties were inoculated simultaneously.

Infected seeds (at least 100 infected grains) were sown in the phytopathological field of SibRIPP&B – Branch of IC&G SB RAS, located in Michurinsky settlement, Novosibirsk region. Soil treatment included spring harrowing, nitrogen fertilization, and cultivation. Trials were established on fallow land. The optimal timeframe, with the soil temperature of 11.5–16.5 °C at a depth of 5–8 cm, which is favorable for both seed and pathogen germination, was chosen for sowing infected seeds.

**Loose smut resistance classification** of the tested samples was performed using the VIR scale (Sanin et al., 2008) by counting healthy and diseased spikes and calculating the infection percentage during the full heading to early ripening stages. When determining the race composition of the loose smut population, resistance classification was based on the international scale: R standing for resistance (infection up to 10 %); S for susceptibility (infection above 10 %).

**Evaluation of lines for agronomic traits.** To assess agronomically valuable traits, a structural analysis of the barley breeding lines was conducted in the autumn of 2022–2023. The traits evaluated included plant height, fertile tillers per plant, spike length, number of grains per spike, and 1,000 grain weight. The evaluation was carried out according to the Methodology of the state variety testing of agricultural crops (1985).

**Fig. 1.**
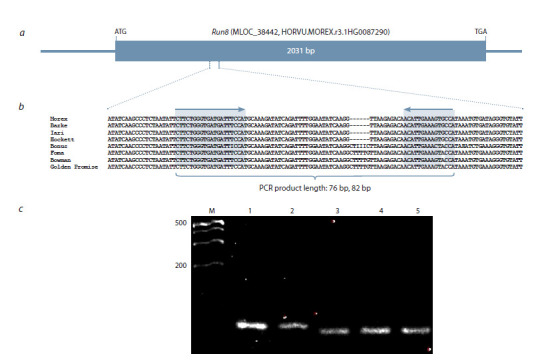
Structural organization of the Run8 gene (a), multiple alignment of nucleotide sequences in the gene region containing a 6 bp insertion/deletion, for which the DNA marker was developed (b), and results of marker testing in barley varieties with known resistance genes to loose smut: Raushan Run8, Run15 (1), Elf Run8 (2), Jet Run3, Run6 (3), Keystone Run6 (4), Grace as a susceptibility standard (5). Primer annealing sites are highlighted in gray, and the expected lengths of PCR fragments for the marker are indicated (c).

**Development of a molecular marker for the Run8 gene. **The molecular marker Hor7050 for the Run8 gene was developed based on the nucleotide sequence MLOC_38442 (HORVU.MOREX.r3.1HG0087290, HORVU1Hr1G087050) (Zang et al., 2015) (Fig. 1a). This sequence was used as a reference in the barley pangenome database (Jayakodi et al., 2024) available on the GrainGenes web page (https://wheat.pw.usda.gov/GG3/), and, as a result, nucleotide sequences of the Run8 gene were found in eight barley samples (Barke, Bonus, Bowman, Foma, Golden Promise, Hockett, Igri, Morex). Multiple sequence alignment was performed using the Multalin v5.4.1 software (Corpet et al., 1988), which revealed polymorphisms among different alleles of this gene. In addition to single nucleotide substitutions, a six-nucleotide deletion was identified in four of the eight varieties. To genotype this deletion, a pair of primers was selected using the IDT PrimerQuest™ (http://eu.idtdna.com/PrimerQuest/Home), with annealing sites flanking the identified deletion (Fig. 1b). Testing the developed marker on barley samples carrying the known loose smut resistance genes showed that electrophoretic analysis in 5 % HR agarose gel made it possible to distinguish the Run8 gene alleles. In resistant barley samples carrying the Run8 gene, the target amplification fragment was 82 bp, and in the susceptible ones 76 bp (Fig. 1c).

By comparing the genotyping data with the resistance of the lines to loose smut, the diagnostic efficiency of the marker was evaluated. It was defined as the proportion of correct test results in the total number of results (i. e., the sum of true positive and true negative results divided by the total number of results).

**Genotyping of lines for the Run6 and Run8 genes.** DNA was extracted from leaves at the tillering stage collected from five plants per line using the method described earlier (Plaschke et al., 1995). DNA was analyzed using the microsatellite marker EBmac0541 linked to the Run6 gene (Menzies et al., 2010) and the intragenic marker Hor7050 developed for the Run8 gene developed in the present paper (Table 1). Polymerase chain reaction (PCR) with the Hor7050 marker was performed in a 20 μl reaction mixture containing 100 ng of DNA template, 1X PCR buffer (67 mM Tris-HCl, pH 8.8, 1.5 mM MgCl2, 0.01 % Tween 20, 18 mM (NH4)2SO4), 0.2 mM of each dNTP, 0.25 μM of each forward and reverse specific primer, and 1 unit of Taq DNA polymerase (Helicon, Moscow). PCR with the EBmac0541 marker was performed using the BioMaster HS-Taq PCR-Color (2×) kit (Biolabmix, Novosibirsk). Amplification with the specified primers was carried out under the conditions as follows: initial denaturation for 1 min 50 s at 94 °C; denaturation for 30 s at 94 °C; primer annealing for 30 s at 50 °C (Hor7050 marker) or 55 °С (EBmac0541 marker), polymerization for 45 s at 72 °С, number of cycles – 45, and final elongation for 5 min at 72 °С. PCR products were separated in 5 % HR agarose gel HR Agarose PCR Grade, HydraGen, NJ, USA) in a horizontal chamber for 3–4 hours at 7 V/cm. UV imaging and gel analysis were performed using the Gel Doc™ XR+ System (Bio-Rad Laboratories, Inc., Hercules, CA, USA).

**Table 1.**
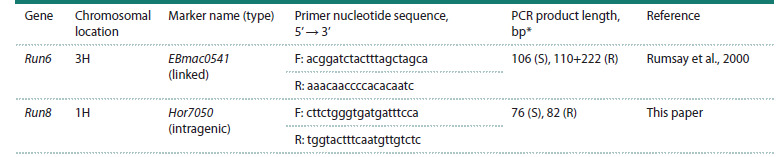
DNA markers used in this paper for genotyping barley lines for the Run6 and Run8 genes * Presented here are the PCR product lengths characteristic of barley samples susceptible (S) and resistant (R) to loose smut.

## Results

A characteristic feature of the 2022–2023 growing seasons was insufficient moisture in May. In 2022, the average daily air temperature in May was +15.4 °C, which is 4.5 °C above the long-term average. In May 2023, although the average monthly temperature was close to the long-term norm, the distribution of heat across decades was uneven. In the second decade, the air temperature was 2.3 °C below the long-term average, while in the third decade it was 3.4 °C above that. The average soil temperature at the depth of 5–8 cm during sowing was 14 °C, which is favorable for the infection process of smut fungi during seed germination. The average air temperatures from May to August were as follows: 15.3, 17.3, 18.5, 16.6 °C in 2022; 11.8, 19.0, 21.6, 17.8 °C in 2023.

In 2022, the total seasonal precipitation was 153.8 mm, which is 30 % as low as the long-term average. In 2023, it was 204 mm, of which 173.8 mm fell in the third decade of July and the second and third decades of August

**Differentiation of the loose smut pathogen U. nuda.** Based on the reaction of the empirical set of differential varieties, it was established that the loose smut population in 2022–2023 was represented by race 1, which does not infect the Keystone variety. Analysis of spore samples using the genetic test set with known resistance genes revealed the presence of biotypes virulent to varieties Trebi (Run1 gene), OAC-21 (Run9 and Run10), and Moskovsky 2 (Run15). Thus, the virulence formula of race P1 is 1 – 1.9.10.15 (Supplementary Tables S1 and S2)1.

Supplementary Materials are available in the online version of the paper: https://vavilov.elpub.ru/jour/manager/files/Suppl_Orlova_Engl_30_3.pdf


**Selection of breeding lines using the molecular marker for the Run8 gene and assessment of their resistance to loose smut.** The differences between the Run8 gene alleles in Elf and Tanai varieties were identified using the Hor7050 marker. A PCR product of 82 bp was obtained on the Elf DNA, and 76 bp on Tanai DNA. Due to the presence of polymorphism between the parental varieties, this marker was used for genotyping 84 F5 breeding lines derived from the Elf × Tanai hybrid population. As a result, 18 lines were identified as having inherited the Run8 gene from the Elf variety.

According to artificial inoculation data from 2021, among the 18 selected breeding lines carrying the Run8 gene from Elf variety, high resistance to loose smut was confirmed in eight lines as follows: 12, 14, 25, 32, 45, 49, 78, and 79. Two lines, specifically 42 and 65, were classified as practically resistant (infection of less than 5 %). Thus, resistance to U. nuda was confirmed by phytopathological methods in 55.6 % of the lines, while the remaining lines showed varying degrees of susceptibility, from weak to strong (Table 2). The diagnostic efficiency of the Hor7050 marker was 0.56.

**Table 2.**
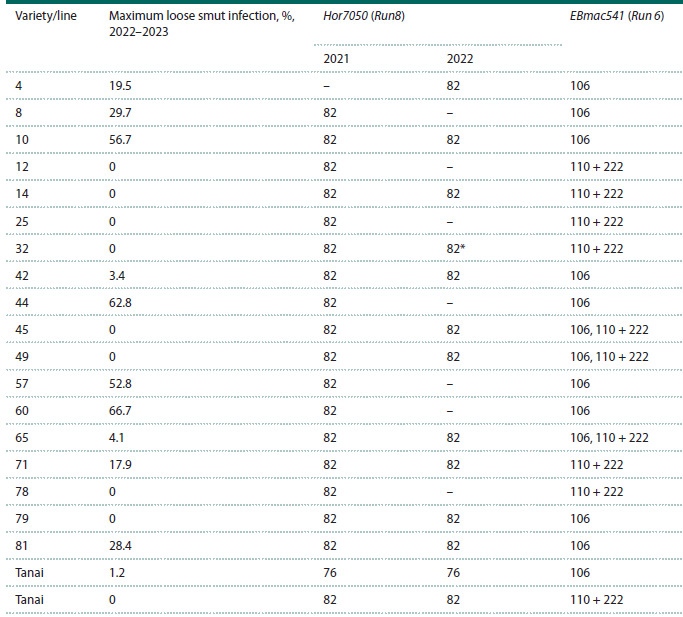
Resistance to loose smut in breeding lines selected from the Elf × Tanai cross and genotyping data for these lines using molecular markers for the Run8 and Run6 genes Note. “–“ indicates that no analysis was performed; * indicates that the allele could not be definitively identified as homozygous or heterozygous

**Fig. 2.**
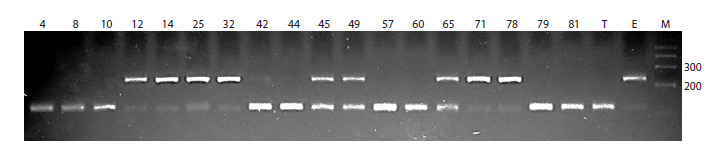
Genotyping results for the selected lines obtained using the microsatellite marker EBmac541. Numbers above indicate line numbers, T stands for Tanai, E for Elf, and М is the 100 bp size marker (bp lengths of length marker fragments are shown on the side).

In 2022, the selected F6 generation lines were additionally genotyped using the Hor7050 marker, which confirmed the presence of the Run8 gene in resistant lines 14, 32, 42, 45, 49, 65, and 79. Unlike the Elf variety, which demonstrated stable resistance to loose smut over three years, not all selected lines carrying the Run8 gene from this variety were resistant to the disease. Lines 4, 8, 10, 44, 57, 60, 71, and 81 showed infection levels ranging from 17.9 to 66.7 %. Since the pedigree of the parental variety Tanai includes the Jet variety known as a carrier of the Run6 gene, the selected lines were genotyped using the microsatellite marker EBmac541 linked to the Run6 gene (Fig. 2).

Out of the 18 analyzed lines, nine inherited both alleles of the EBmac541 marker from the Tanai variety (lines 4, 8, 10, 42, 44, 57, 60, 79, and 81), six from the Elf variety (lines 12, 14, 25, 32, 71, and 78), and three lines exhibited marker heterozygosity (lines 45, 49, and 65). By comparing the genotyping data with the resistance of the studied lines to loose smut, it was found that among the ten resistant lines (infection rate from 0 to 4.1 %), eight carried the EBmac541 marker allele from the Elf variety in either homozygous or heterozygous state. Among the eight susceptible lines (infection rate from 17.9 to 66.7 %), only line 71 carried the Elf allele of the marker, while the others inherited the allele from Tanai. Based on these results, the diagnostic efficiency of the EBmac541, marker turned out to be 0.83.

**Evaluation of selected lines for agronomic traits.** To assess agronomically valuable traits, a structural analysis was conducted in the autumn of 2022–2023 for the resistant barley breeding lines carrying the Run8 gene. Both biometric and productivity-related traits were evaluated. The parental variety Elf, carrying the Run8 gene, was used as a control.

All tested lines outperformed the control variety Elf in plant height. Lines 32, 42, 65, 78, and 79 also surpassed the paternal form Tanai, with average heights over two years ranging from 70.0 to 75.3 cm. The remaining lines showed an intermediate performance between the parental forms. Spike length is known to positively influence productivity, as it has a high correlation with the number of grains per spike (Naumova, 2021). In the selected lines, spike length ranged from 5.9 (line 12) to 7.8 cm (line 65). Lines 32, 65, and 79 significantly exceeded both parental forms in spike length and number of grains per spike. Lines 12, 42, 49, and 78 showed low spike productivity (Table 3).

**Table 3.**
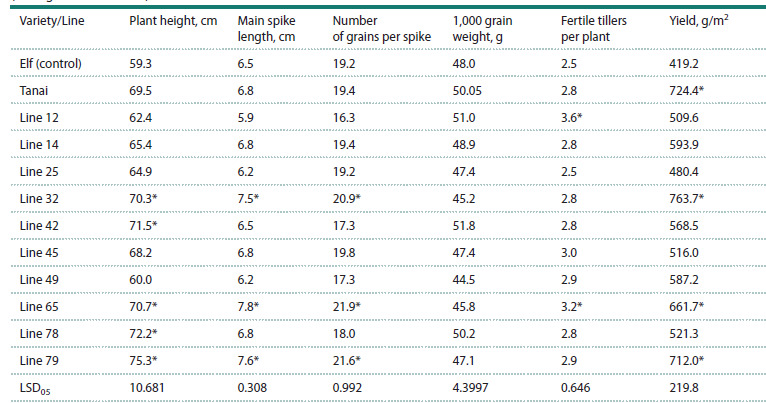
Main agronomic traits of breeding lines from the Elf × Tanai cross, F5 generation (average for 2022–2023) Indicates statistically significant differences compared to the parental variety Elf.

Grain size is another important yield component. In 2022, the highest 1,000 grain weight of 54.5 g was observed in line 49 compared to 51.1 g in Elf. Over two years, grain sizes comparable to Tanai were recorded in lines 12, 42, and 78 reaching 51.0, 51.8, and 50.2 g, respectively.

The number of fertile tillers per plant in lines 12 and 65 was 1.4 to 1.3 times as high as that of the control variety Elf reaching 3.6–3.2 fertile stems per plant.

Based on the two-year data, three productive lines were identified, namely 32, 65, and 79, which significantly outperformed the control variety Elf in yield. In line 65, yield varied by year from 809.7 g/m2 in 2022 to 513.7 g/m2 in 2023. Line 32 showed the most stable yield, with 756.0– 771.3 g/m2 across years, and on average over two years, it exceeded the best parental form, Tanai. Line 79 stood out for its earliness and productivity.

Thus, as a result of molecular genetic analysis, 18 breeding lines carrying the Run8 loose smut resistance gene were selected from the Elf × Tanai (F5) hybrid combination. Resistance in 10 of these lines was confirmed by phytopathological testing. Lines 12, 14, 25, 32, 42, 45, 49, 65, 78, and 79 were classified as highly or practically resistant barley genotypes. Based on structural analysis data, the standout hybrid lines 32, 65, and 79 were transferred to breeding nurseries for further evaluation of agronomically important traits

## Discussion

Due to the specific life cycle of the basidiomycete U. nuda (Jens.) Roster, phenotyping for resistance to the disease it causes, i. e. loose smut, is a lengthy and labor-intensive process. Therefore, diagnostic molecular markers gain special relevance in breeding for resistance to the disease. However, to date, many Run genes that control resistance to loose smut have not yet been mapped in the barley genome (Abo-Elyousr et al., 2022). Among the genes with known chromosomal locations, only two, namely Run6 and Run8, have been mapped to chromosomes 3H and 1H, respectively (Menzies et al., 2010; Zang et al., 2015). Precise mapping has traced the latter back to a specific nucleotide sequence coding for a protein kinase (Zang et al., 2015). Long-term

testing of varieties with identified resistance genes has shown that Run6 and Run8 were effective against loose smut races in Western Siberia. In the present paper, the source of the Run8 gene, namely the Elf variety, was used as a donor in the development of loose smut-resistant lines. To select resistant lines, the Hor7050 molecular marker was developed based on the nucleotide sequence of the Run8 gene identified by W. Zang et al. (2015). When comparing the amino acid sequences of protein kinases predicted from Run8 gene sequences, it was shown in the present paper that the group of loose smut-resistant samples was characterized by 15 unique amino acid substitutions. These substitutions were caused by single nucleotide polymorphisms (SNPs), the detection of which by routine PCR is labor-intensive and requires the development of specific primers, probes, or selection of restriction endonucleases. In this paper, a common six-nucleotide deletion was identified by comparing Run8 gene sequences from the barley pangenome database. This deletion results in the loss of two amino acid residues in the protein kinase. When comparing Run8 protein sequences, the deletion of two amino acid residues was found in loose smut-susceptible barley samples, whereas the presence of these two amino acids was observed in both resistant and susceptible samples (Zang et al., 2015). Thus, the marker developed specifically for this deletion only has partial diagnostic efficiency, since not all barley samples carrying the six-nucleotide insertion will be resistant to loose smut. However, it can be used to track the inheritance of the Run8 allele from a known donor. The total of 18 barley lines carrying the Run8 gene from the resistant donor Elf were selected using this marker. However, unlike the Elf variety, some of the selected lines showed susceptibility to race 1 of loose smut, as identified using differential varieties. Since the second parent, Tanai, has the Jet variety in its pedigree, which is known as a donor of the Run6 gene, a hypothesis was posed that the resistant lines might carry the Run6 gene from the Tanai variety in addition to Run8 from Elf. To test this hypothesis, the selected lines were genotyped using the EBmac0541 marker linked to the Run6 gene (Menzies et al., 2010). However, the analysis showed that most of the lines demonstrating resistance to loose smut inherited the EBmac0541 marker from Elf, rather than Tanai. Lines 4, 8, 10, 44, 57, 60, and 81, which inherited the EBmac0541 marker from Tanai, were susceptible to loose smut, despite carrying the Run8 gene from Elf. The only line that carried the EBmac0541 marker from Tanai and was resistant to loose smut was line 79. Since EBmac0541 is linked to Run6 gene, the discrepancy between the marker allele and the loose smut resistance trait may be explained by recombination between the marker used for genotyping and the gene controlling the trait. The analysis performed makes it possible to assume that the Elf genome may also contain Run6 gene effective against race 1 of loose smut in addition to Run8. Further analysis of Run8 nucleotide sequences in known resistance donors, as well as the co-segregation observed in this paper between the EBmac0541 marker from Elf and resistance to loose smut, will help clarify the roles of Run6 and Run8 in resistance to pathogen races prevalent in Western Siberia. Phytopathological assessment and productivity evaluation of the selected lines over two years made it possible to identify three promising breeding lines resistant to loose smut and significantly outperforming the control variety Elf in yield. These selected lines have been transferred to breeding nurseries for further evaluation of agronomically valuable traits.

## Conclusion

In the present paper, a diagnostic intragenic molecular marker was developed based on the nucleotide sequence of the Run8 gene. Its application, in combination with the microsatellite marker EBmac0541 linked to the Run6 gene made it possible to identify an association between the EBmac0541 allele from the Elf variety and resistance of barley hybrids to loose smut. As a result of the research, promising breeding lines were selected that are comparable in productivity to the original varieties

## Conflict of interest

The authors declare no conflict of interest.
